# Optimisation of asymmetric flow field-flow fractionation for the characterisation of nanoparticles in coated polydisperse TiO_2_ with applications in food and feed

**DOI:** 10.1080/19440049.2016.1239031

**Published:** 2016-10-24

**Authors:** J. Omar, A. Boix, G. Kerckhove, C. von Holst

**Affiliations:** ^a^ European Commission, Joint Research Centre (EC-JRC), Geel, Belgium

**Keywords:** Asymmetric flow, field-flow fractionation, titanium dioxide, polydisperse material, nanoparticles

## Abstract

Titanium dioxide (TiO_2_) has various applications in consumer products and is also used as an additive in food and feeding stuffs. For the characterisation of this product, including the determination of nanoparticles, there is a strong need for the availability of corresponding methods of analysis. This paper presents an optimisation process for the characterisation of polydisperse-coated TiO_2_ nanoparticles. As a first step, probe ultrasonication was optimised using a central composite design in which the amplitude and time were the selected variables to disperse, i.e., to break up agglomerates and/or aggregates of the material. The results showed that high amplitudes (60%) favoured a better dispersion and time was fixed in mid-values (5 min). In a next step, key factors of asymmetric flow field-flow fraction (AF4), namely cross-flow (CF), detector flow (DF), exponential decay of the cross-flow (CF_exp_) and focus time (Ft), were studied through experimental design. Firstly, a full-factorial design was employed to establish the statistically significant factors (*p* < 0.05). Then, the information obtained from the full-factorial design was utilised by applying a central composite design to obtain the following optimum conditions of the system: CF, 1.6 ml min^–1^; DF, 0.4 ml min^–1^; Ft, 5 min; and CF_exp_, 0.6. Once the optimum conditions were obtained, the stability of the dispersed sample was measured for 24 h by analysing 10 replicates with AF4 in order to assess the performance of the optimised dispersion protocol. Finally, the recovery of the optimised method, particle shape and particle size distribution were estimated.

## Introduction

Nanotechnology is an important element to foster economic growth worldwide due to its widespread use; it is applicable in many disciplines and combines both science and engineering (Baalousha & Lead 2012; Chekli et al. 2015). Due to this extensive usage of nanomaterials, the European Commission (EC) published in 2011 the Recommendation on the definition of nanomaterials as:

a natural, incidental or manufactured material containing particles, in an unbound state or as an aggregate or as an agglomerate and where, for 50% or more of the particles in the number size distribution, one or more external dimensions is in the size range 1–100 nm.

(Commission Recommendation 2011/696/EU)

The proper implementation of this definition triggered the need for developing and validating characterisation and quantification methodologies for nanomaterials (Roebben et al. 2014). Presently, there is no single reliable method to fulfil all the requirements of the EC definition (Roebben et al. 2014), thus the combination of several techniques is frequently required.

Titanium dioxide (TiO_2_) is one of the most widely employed nanomaterials. It is used in paints, cosmetics (due to its UV radiation-blocking capacity), as an additive (sensory additives: food colorants and/or ‘anti-caking’ agents), construction and building materials because it is considered insoluble, stable, non-reactive, low cost and non-flammable (Contado & Pagnoni 2008; Chekli et al. 2015). The characterisation of polydisperse materials consisting of agglomerates and/or aggregates, as well as constituent particles, is very challenging. The studied polydisperse TiO_2_ is part of the Titanium Dioxide series from the EC’s Joint Research Centre (JRC) Nanomaterials Repository (JRC 2014) that meets the need for nanosafety research purposes and has been characterised in the frame of several European projects using various analytical techniques (Rasmussen et al. 2014). Given the potential of field-flow fractionation as a robust technique to separate particles according to their size and that fractionation methods have not been applied to the characterisation study mentioned above, the aim of this study is to optimise an asymmetric flow field-flow fractionation (AF4) method that can contribute to the physicochemical characterisation of TiO_2_.

AF4 is a very useful technique for the characterisation of such polydisperse materials due to its robust size-based separation capacity (von der Kammer et al. 2005; Contado & Pagnoni 2008; Samontha et al. 2011). Moreover, it can be coupled to different detectors, such as inductively coupled plasma-mass spectrometry (ICP-MS), dynamic light scattering (DLS), multi-angle light scattering (MALS), and ultraviolet-visible spectrophotometer (UV-Vis) to allow for a thorough characterisation of the size of the nanoparticles. Another possibility is to collect the fractions of the eluting material from the AF4 and to analyse them offline using SEM or TEM (von der Kammer et al. 2011).

According to Baalousha and Lead (2012), different techniques can result in a high variability of the measured sizes and the result of analysis can also be affected by other factors such as the experimental conditions and the aggregation state of the sample. Moreover, the lack of suitable reference materials in the nano-range for the characterisation of a polydisperse material makes it difficult to cross-correlate and validate new methods (Roebben et al. 2014). One certified reference material for non-coated TiO_2_ (SRM1898) is obtainable from NIST (Certificate of Analysis; NIST 2012), but this material does not cover the range of sizes of a polydisperse material.

Appropriate sample preparation is crucial for a successful AF4 separation (Wagner et al. 2015), and the consequent characterisation of nanoparticles. An optimised dispersion protocol, including the selection of an appropriate solvent, ensures, on the one hand, that the material is brought to the smallest dispersible unit possible and, on the other hand, that the sample is stable in solution, i.e., it does not sediment or agglomerate easily. The examination of the test material before and after sample preparation was supported by SEM.

In this study, the sample preparation and conditions of the AF4 method were optimised using experimental design. The main advantage of the experimental design is that a reduced number of experiments are required and the interactions among the experimental factors can be studied (Yuangyai & Nembhard 2015). Alternatively, the ‘one factor at a time approach’ is often employed in the sample preparation by varying the power of the sonicator, using different kinds of sonication (ultrasonic bath, probe, cup), applying titrations, filtering, sedimentation, etc. (Geiss et al. 2013; Lopez-Heras et al. 2014; Antonio et al. 2015). By applying this concept, a starting combination of factors is selected and varied one at a time, whereas all the other factors are kept constant. While this concept is often used in method optimisation, it shows some limitations by not allowing for an easy identification of interactions between experimental factors regarding their influence on the selected response. Likewise, curvilinear effects of these factors can hardly be addressed. These underlying reasons explain the use of experimental design in this work.

The purpose of the study was to propose an approach based on the use of experimental design for the optimisation of the AF4 method to characterise polydisperse-coated TiO_2_. The availability of this method is considered as an important prerequisite to investigate the nano-characteristics of TiO_2_ containing food and feed additives. The work included the optimisation of the required dispersion protocol of the test samples and the AF4 conditions.

## Materials and methods

### Chemicals and samples

Titanium dioxide (TiO_2_) from the JRC Nanomaterials Repository JRCNM0-1004a (former code NM-104) was employed in this study. An extensive batch of TiO_2_ materials was previously characterised using different techniques as summarised in the JRC report (Rasmussen et al. 2014). The material is described as rutile, thermal hydrophilic, a solid white powder and is in the form of nanocrystals, containing 89% of TiO_2_ and 6.2% aluminium oxide (used for the coating). The primary particle size, which is given by TEM in the JRC report, is 23–27 nm, and the particle size distribution is claimed to be 53% < 100 nm and 12% < 50 nm. However, the nominal value of the analysed material measured by DLS, small-angle X-ray scattering (SAXS) and TEM is 117.8 ± 76.4 nm (Rasmussen et al. 2014), which will be the nominal value used in the optimisation presented in this study.

### Experimental design

The experimental conditions of the dispersion and AF4 procedure were separately optimised by conducting measurements according to an experimental design (Bayne & Rubin 1986). This approach is based on the principle that the experimental conditions are varied following a predefined plan, and a group of experiments is performed accordingly, followed by a statistical assessment of the results of analysis. The purpose of the statistical evaluation is to identify the conditions in which the target response is optimised. In this study, the experimental conditions referred to the details of the dispersion and AF4 protocols respectively, while the response was the measured size (nm) of the particles, eluting at 65 min when the cross-flow was finished, and all the remaining material in the separation chamber was eluted. According to the JRC report (Rasmussen et al. 2014), 200 nm should be the biggest particle size found in this material; thus, the response was considered optimised when the particle size measured at 65 min was close to 200 nm, whereas the material eluted after the 65 min was not considered relevant for the purpose of this study.

Once the best solution for the dispersion protocol was identified, experiments were carried out to optimise key conditions of the dispersion protocol, which were the sonication time and amplitude. A specific design of these experiments was applied to fit a second-order model to the experimental data, which described the dependence of the measured particle size upon these two factors and to estimate the coefficient of the corresponding prediction equation. The model contained linear terms of these factors, a term specifying an interaction of both factors and a quadratic term. By including a quadratic term, the model was able to describe situations in which the measured particle size passed through an optimum obtained at specific combinations of these two factors. A central composite design (CCD) was applied (Bayne & Rubin 1986) to establish the levels of these factors at which the experiments were conducted. CCDs are particularly suitable when dealing with second-order models and have the advantage that the required number of experiments and the correlation between estimated coefficients of the model are minimised (Bayne & Rubin 1986). Here, the factors were varied across five levels ranging from 19% to 61% for the sonication amplitude and from 1.5 to 11.5 min for the sonication time. The design comprised 14 experiments including six replicates for the central point, i.e., at 40% for the sonication amplitude and 6.5 min for the sonication time, as shown in Table 1. The results from the replicates were required to estimate the analytical error of the measurement, which allowed for a check of the significance of the measured effects. Finally, the model was used to establish a response surface plot in which the measured particle size was plotted against the two factors.

**Table 1.** Sample preparation by focused ultrasound: central composite design and the results of analysis expressed in terms of particle size (nm) at minute 65 when the cross-flow of the separation method is finished. The experimental design was based on sonication amplitude (%) and sonication time (min). The target value for the response was 200 nm as given in the JRC report.

Trial number	Levels	Amplitude (%)	Sonication time (min)	Response (nm)
1	(–√*α*), (0)	19	6.5	309
2	(0), (0)	40	6.5	268
3	(+1), (–1)	55	3	223
4	(+1), (+1)	55	10	254
5	(0), (0)	40	6.5	233
6	(0), (–√*α*)	40	1.5	321
7	(0), (0)	40	6.5	225
8	(–1), (+1)	25	10	290
9	(0), (+√*α*)	40	11.5	269
10	(–1), (–1)	25	3	256
11	(0), (0)	40	6.5	237
12	(+√*α*), (0)	61	6.5	203
13	(0), (0)	40	6.5	229
14	(0), (0)	40	6.5	240

Note: *α*, Number of factors to be studied, i.e., two.

For the optimisation of the AF4 method, four key instrumental factors were selected, namely: cross-flow (CF), detector flow (DF), focusing time (Ft), and exponential decay of the cross-flow (CF_exp_). These variables were selected because in previous studies (Loeschner et al. 2013; Lopez-Heras et al. 2014; Mudalige et al. 2015) CF and Ft were defined as key factors to be controlled during particle size distribution measurements. Furthermore, CF is one of the main factors controlling the distribution of particles near the membrane and, consequently, their separation. Also, the focusing step is essential for minimising peak broadening during sample injection, avoiding particle losses and also controlling aggregation.

First, a full-factorial design (FFD) was applied in which all four factors were varied at two levels. Also, a central point was included in the design at which all factors were kept at the mean value of the two levels to estimate the analytical error of the experiments. The main purpose was to identify those factors that have a significant effect on the measured particle size. The FFD also allowed for the evaluation of an interaction of two factors, which is obvious, if the effect of one factor depends on the level of another factor. The FFD required 22 experiments including six replicates of the central point covering a factor space in the ranges shown in Table 2. Next, a CCD was applied to two statistically significant factors, as shown in Table 3, and following the same procedure as previously described. Again, the total number of experiments was 14 and both factors were varied across five levels, namely from 1 to 2.45 ml min^–1^ for CF and from 0.1 to 0.4 ml min^–1^ for DF.

**Table 2.** AF4 system: Factors studied by a Full Factorial Design (FFD) for the optimisation of the AF4 system with the JRCNM0-1004a material at three levels which show the studied range. The instrumental factors are: cross-flow (CF), detector flow (DF), focusing time (Ft) and the exponential decay of the cross-flow (CF_exp_). The statistical assessment showed that exclusively CF and DF turned out to be significant and therefore only these parameters were included in the CCD.

	FFD levels
CF (ml min^–1^)	0.5–1.5–2.5
DF* (ml min^–1^)	0.2–0.35–0.5
Ft (min)	3–5–7
CF_rate_ (%)	0.2–0.6–1

Note: CF and DF factors employed in the CCD.

**Table 3.** AF4 system: central composite design using the statistically significant factors cross-flow (CF) and detector flow (DF) identified in the previous full-factorial design (FFD) and the results of analysis expressed in terms of the particle size (nm) at minute 65. The experiments were performed with the JRCNM0-1004a material.

Trial number	Levels	CF (ml min^–1^)	DF (ml min^–1^)	Response (nm)
1	(0), (–√*α*)	1.6	0.1	78.8
2	(0), (0)	1.6	0.275	151.5
3	(+√*α*), (0)	2.45	0.275	116.3
4	(0), (0)	1.6	0.275	146.5
5	(–√*α*), (0)	0.75	0.275	268.2
6	(0), (+√*α*)	1.6	0.45	228.8
7	(0), (0)	1.6	0.275	144.0
8	(+1), (–1)	2.2	0.15	85.1
9	(0), (0)	1.6	0.275	142.8
10	(–1), (+1)	1.0	0.4	367.0
11	(+1), (+1)	2.2	0.4	156.6
12	(0), (0)	1.6	0.275	141.4
13	(–1), (–1)	1.0	0.15	121.4
14	(0), (0)	1.6	0.275	135.3

All experiments of the various experimental designs were carried out in a random sequence. The commercial software packages The Unscrambler (The Unscrambler X®, v10.3, CAMO, Trondheim, Norway) and Statistica 10 (Stat Soft Inc. USA) were used for planning and statistical assessment of the experimental designs.

### Preparation of samples

A standard dispersion of 0.5 mg ml^–1^ was prepared by weighing accurately 15 mg of JRCNM0-1004a and mixing it with 30 ml 0.1% aqueous sodium pyrophosphate decahydrate (NaPP, Sigma-Aldrich, Diegem, Belgium) solution previously filtered through a 0.2 µm hydrophilic polypropylene membrane filter (PALL Life Sciences, Tienen, Belgium). The solution was then sonicated, keeping the vial refrigerated in a water/ice bath through probe ultrasonicator, using a microtip (Qsonica, Q700 sonicator, Newtown, CT, USA). As stated by Taurozzi et al. (2011), a pulsed operation mode was chosen in all cases (5 s on, 5 s off) in order to help the ice/water bath maintain its temperature and to avoid heating the suspension that would lead to undesirable side-effects. The final conditions used in the sample preparation were 60% sonication amplitude for 5 min, which were the optimum conditions obtained, as will be explained below.

The samples from the experiments that optimised the sample preparation protocol were analysed with an AF4 method using the following experimental conditions recommended by the instrument manufacturer and which were identical in all experiments. Cross-flow: 1.5 ml min^–1^; detector flow: 0.35 ml min^–1^; focus time: 5 min; injection flow: 0.2 ml min^–1^; and injection volume: 20 µl of sample. As recommended in the literature (Contado & Pagnoni 2008; von der Kammer et al. 2011), it is important to ensure that the composition of the solvent used in the dispersion protocol equals the composition of the carrier used for the AF4 to keep the particles dispersed.

### Instrumentation

An asymmetric flow field flow fractionation (AF4) system (Postnova Analytics, Landsberg, Germany), composed of: PN7140 solvent organiser; PN 7205 UV disinfector; PN7520 solvent degasser; two PN 1130 isocratic pumps; AF 2000MT field-flow fractionation; PN 5300 autosampler; PN 4020 oven equipped with a regenerated cellulose membrane of 10 kDa molecular weight cut-off; and a spacer of 350 µm was used in this study. The AF4 system was coupled to: (1) a Shimadzu Diode Array Detector (DAD) (SPD-M20AV UV/VIS detector, Tokyo, Japan) with a wavelength range of 190–800 nm; (2) DLS system (Zetaziser Nano, Malvern, UK); and (3) PN 3621 MALS detector (Postnova Analytics).

The results from the DLS detector were used as a response for all the designs because the particle size at an elution time of 65 min can be straightforwardly obtained from the fractograms. It has to be considered that DLS has some limitations, such as the fact that it considers all particles as spherical, interferences are not easily identified, etc. (Brar & Verna 2011; Tomaszewska et al. 2013). Moreover, it is known that by means of the DLS, the hydrodynamic diameter obtained is typically larger than the core of the particle because it also considers the hydration shell (Taurozzi et al. 2013; Tomaszewska et al. 2013). Thus, the developed sample preparation protocol will ensure that the dispersed material will always be below 200 nm, which is the maximum particle size given in Rasmussen et al. (2014).

The instrument control and data analysis of the AF4 were performed by the AF2000 Control software (Postnova Analytics); the instrument control and data analysis of the DAD were carried out by LabSolution platform (Shimadzu, Tokyo, Japan). The instrument control and data analysis of the DLS were conducted by Zetasizer software.

**Figure 1.**
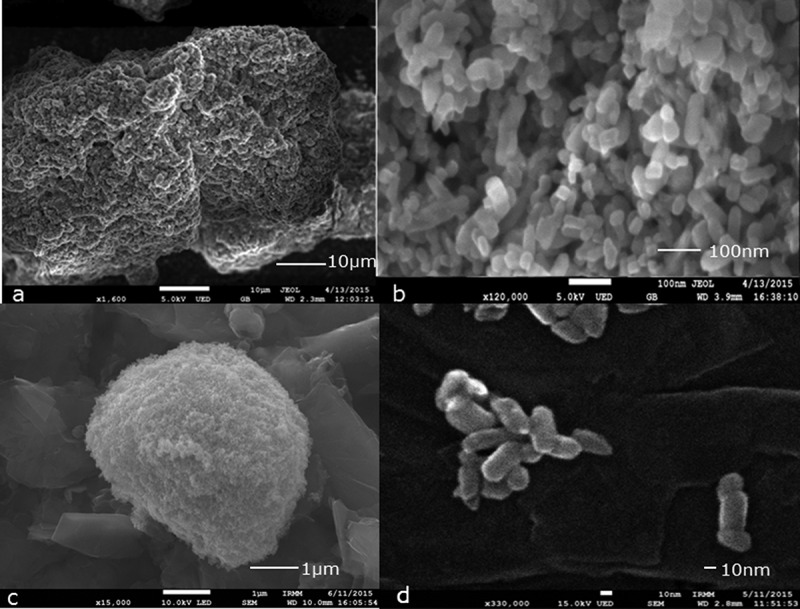
Illustrative images of JRCNM0-1004a material obtained by scanning electron microscopy as such and after different treatments: (a) deposited solid test material on a carbon stub; (b) the same experiment as in (a) but with higher magnification; (c) test material dispersed in NaPP 0.1%; and (d) test material after sonication at 60% during 5 min.

A field emission-scanning electron microscope (FE-SEM, JEOL JSM-7800F) was used to observe the JRCNM0-1004a material at different sample preparation stages: solid material (as received), mixed with the solvent NaPP 0.1% and probe sonicated. The material (white powder) was carefully sprinkled from a spatula onto a carbon JEOL SEM mount, coated with tacky colloidal carbon for its preliminary observation (Figure 1(a): magnification 1600, 5 kV accelerating voltage, gentle beam mode with upper electron detector and working distance of 2.3 mm; and Figure 1(b): magnification 120,000, 5 kV accelerating voltage, gentle beam mode with upper electron detector and working distance of 4 mm). Next, the material was mixed with the solvent, NaPP 0.1%, for the analysis on the AF4; a 10 µl drop was deposited on top of a carbon stub and was left to dry for 48 h in a fume hood. After this period, new images were acquired to see how the material behaves in solution (Figure 1(c): magnification 15,000, accelerating voltage 10 kV SEM mode with lower electron detector and working distance 10 mm). Finally, the JRCNM0-1004 was mixed with the solvent chosen (NaPP 0.1%) and was probe sonicated at 60% for 5 min; a 10 µl drop was deposited on top of the carbon stub and was left to dry for 48 h in a fume hood before taking images of it in order to evaluate the effect of the probe sonication (Figure 1(d): magnification 330,000 15 kV in SEM mode with the upper electron detector and working distance 2.8 mm).

### AF4 conditions

The following optimised AF4 conditions were obtained: 20 µl of sample (0.5 mg ml^–1^) were injected into the AF4 system; focusing time was 5 min; cross-flow, 1.6 ml min^–1^; detector flow, 0.4 ml min^–^^1^; and exponential decay of the cross-flow was fixed at 0.6. Furthermore, the results showed that after each run a blank analysis must be carried out to avoid carryover effects, which could affect the next sample.

## Results and discussion

### Selection of the solution used in the dispersion protocol and as AF4 carrier

Based on the literature available (Nischwitz & Goenaga-Infante 2012; Guiot & Spalla 2013; Lopez-Heras et al. 2014), we applied different dispersion protocols and carriers for the separation of TiO_2_ nanoparticles. Many parameters, such as ionic strength, surfactants and pH, can affect the interaction between nanoparticles and/or between the membrane and nanoparticles (Lopez-Heras et al. 2014). It has been stated that the presence of an electrolyte in the carrier fluid is necessary because the electrostatic repulsion between the nanoparticles and the membrane is reduced, thus allowing the particles to equilibrate closer to the membrane, which causes longer retention of the analyte and subsequent separation in AF4 (Peters et al. 2014). Therefore, different sample preparations for the TiO_2_ material were tried (e.g., different salts, surfactants); the same solvents were used as carriers for the AF4. The sonication conditions (3 min at 30% amplitude) were the same for all the trials. Five different solvents were tried: (1) water; (2) 0.05% aqueous bovine serum albumin (BSA) (Rasmussen et al. 2014); (3) 0.2% aqueous FL-70 detergent (Nischwitz & Goenaga-Infante 2012); (4) BSA 0.05% as carrier and sample preparation according to Guiot and Spalla (2013); and (5) 0.1% aqueous NaPP. Among the five different dispersants, 0.1% NaPP gave the best particle size distribution following what was stated in Rasmussen et al. (2014). Moreover, the highest peak intensity and best peak shape among the different combinations were also checked (results not shown). Therefore, 0.1% NaPP was used in the optimised sample preparation protocol.

### Optimisation of the focused ultrasound for the sample preparation

Among the sample preparation steps, sonication is a key step for many authors (Contado & Pagnoni 2008; Taurozzi et al. 2011). Some researchers used an ultrasonic bath, getting the ultrasounds across the container into the sample, but ultrasonication using a probe sonicator is a more efficient way of generating ultrasonic cavitation to disperse particles in a liquid (Taurozzi et al. 2011, 2013; Cascio et al. 2014). Therefore, all experiments were conducted with the probe sonicator.

**Figure 2.**
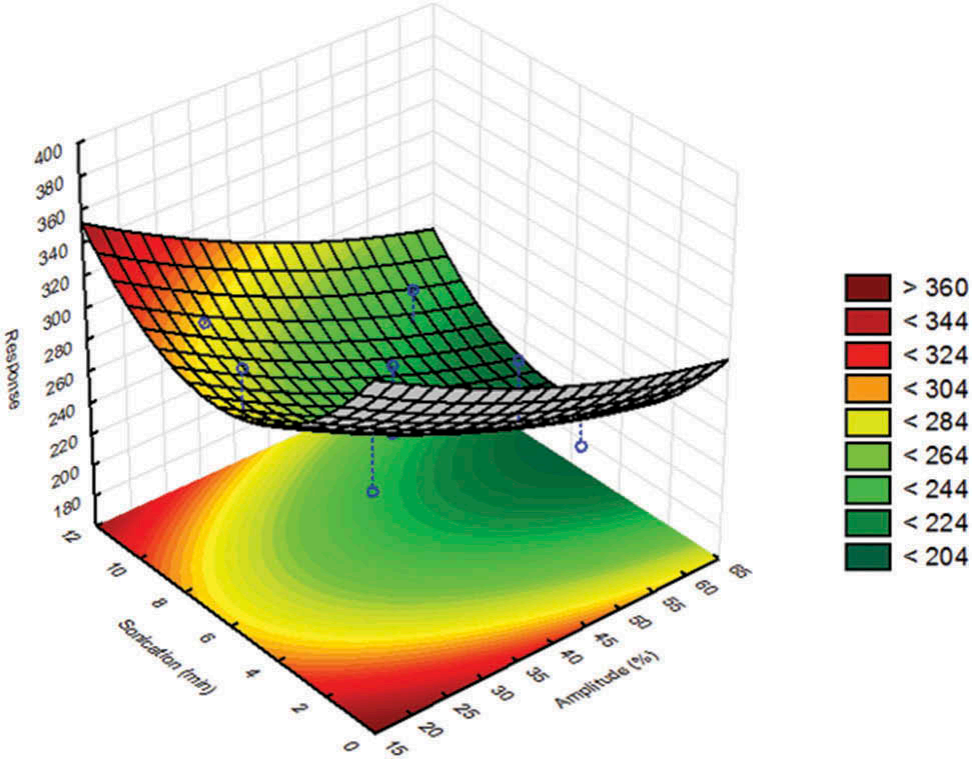
Statistical evaluation of the sample preparation by focused ultrasound: response surface and contour plot of JRCNM0-1004a (TiO_2_) in which particle size (nm) measured at 65 min is plotted against the amplitude/power (19–61%) of the ultrasound system and the sonication time (1.5–11.5 min).

Subsequently, the sonication time and amplitude were optimised by applying the CCD. The results from the statistical analysis of the results from the CCD are shown in Figure 2, where the response of the measured particle size was plotted against these two factors. The region with the measured particle size close to 200 nm was coloured dark green, while the region with larger particles was coloured red. A visual examination of the response surface revealed that the sonication time passed an optimum that was between 5 and 7 min, whereas optimal values for the particle size were obtained when the amplitude was above 50%. Therefore, the amplitude was set at 60% and the sonication time was set at 5 min, which was the lower value of the optimal range required to save time between experiments in the final method protocol for the sample preparation. In addition, the selected conditions for the amplitude contribute to minimising the risk of generating smaller particles.

In examining visually and qualitatively the impact of the sample preparation on the material, several SEM images were acquired at different stages, and the best representatives are shown in Figure 1. Images of such material were obtained by carefully depositing a few particles on the surface of the carbon stub (Figure 1(a, b)). Next, a drop (10 µl) of the sample (JRCNM0-1004a), mixed by hand with the solvent (NaPP 0.1%), was deposited on top of a carbon stub (Figure 1(c)) and dried for 48 h and measured. The aim of this measurement was to qualitatively observe changes of the sample after being in contact with the solvent. Finally, the sample was prepared under the optimised conditions (60% amplitude during 5 min in the probe sonicator), deposited, dried for 48 h and measured by SEM (Figure 1(d)). The visual inspection of the obtained figures showed that the JRCNM0-1004a material (Figure 1(a, b)) is presented in big clusters. After adding the solvent, the clusters become smaller and more rounded, but they are still at the micron scale. By contrast, after the optimised sample treatment, the big clusters are broken into smaller agglomerates/aggregates (Figure 1(d)). This image also shows that the particles of this material have very different sizes and shapes. It has to be considered that the sample preparation required drying of the suspension drop, which can induce additional aggregation (Loeschner et al. 2013).

### Optimisation of the AF4 system for the separation of JRCNM0-1004a TiO_2_ nanoparticles

**Figure 3.**
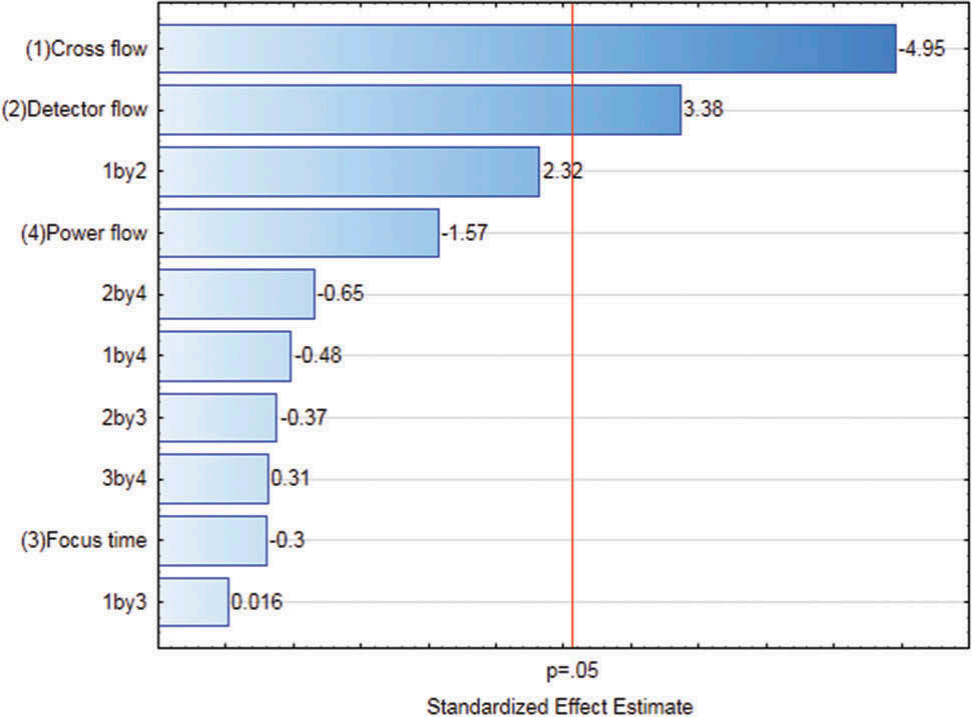
(colour online) Pareto chart of the factors influencing the measured particle size of the AF4 method: the effects of the factors and corresponding interactions were considered significant when their corresponding values were larger than the reference value (*p* = 0.05), indicated by a vertical red line. Interactions were shown by the corresponding number. For instance, ‘1 by 2’ indicated the interaction of cross-flow (1) and detector flow (2). Note: Power flow = CF_exp_.

The results of the statistical assessment of the FFD are shown in a Pareto chart (Figure 3) where the magnitude of the effect of each factor and interaction of these factors are plotted along with a reference level indicating a significance level of 0.05. Effects and interactions were only considered significant if their values were above this reference level. Based on the results shown in Figure 3, only the cross-flow and detector flow were found to be significant, whereas the effects of the remaining two factors and all interactions turned out to be insignificant. Therefore, the dependence of the measured particle size on CF and DF was subjected to further statistical assessment.

**Figure 4.**
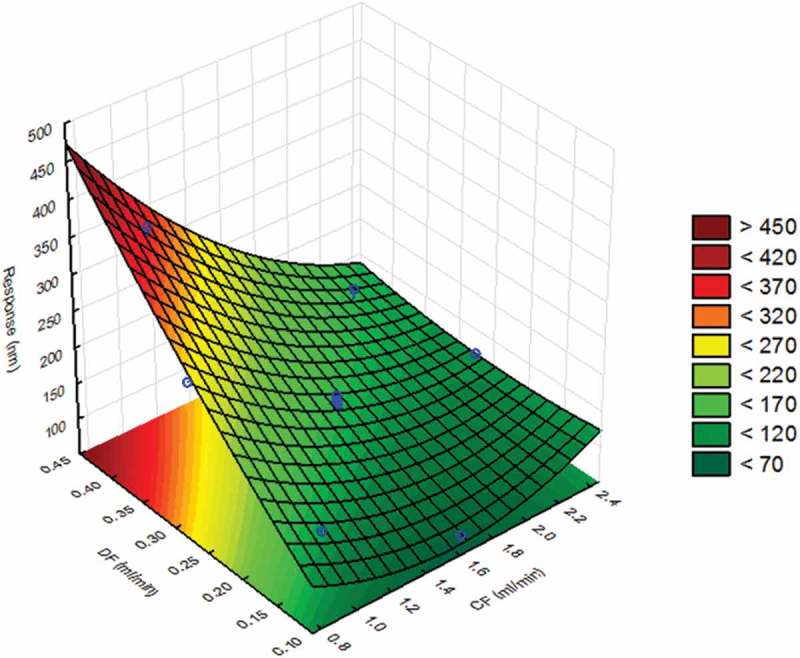
Statistical evaluation of the AF4 system: response surface of JRCNM0-1004a (TiO_2_) in which the particle size (nm) measured at 65 min is plotted against cross-flow (CF, range = 0.75–2.45 ml min^–1^) and detector flow (DF, range = 0.1–0.45 ml min^–1^). A value of 200 nm for the measured particle size was considered as optimum.

Next, an optimisation design (CCD) was performed focusing on CF and DF. In this CCD, these factors were varied across five levels, from 0.75 to 2.45 ml min^–1^ for CF and from 0.1 to 0.45 ml min^–1^ for DF, as shown in Table 3, whereas Ft and CF_exp_ were fixed at 5 min and 0.6% respectively, which were the mean levels of the FFD, as shown in Table 2. Then the coefficients of the second-order model were calculated; the corresponding response surface and contour plot are shown in Figure 4. The region with optimal values for the measured particle size was coloured light green, whereas the region with largely measured particles was dark red and the region with small particles was dark green. A visual inspection of the surface showed that DF values should be above 0.2 ml min^–1^ in order to ensure that the particle size was below 200 nm. Also, it was recommended by the instrument manufacturer to avoid low DF values because the pressure of the system may not be stable enough, thus rendering the system less reproducible. Therefore, this parameter was set at 0.4 ml min^–1^. Furthermore, the response surface indicated that at this DF value the measured particle size depended largely on the CF value. For instance, too large particles above 300 nm could be expected when using CF values below 1 ml min^–1^, whereas at high CF values above 2 ml min^–1^ a particle size of 150 nm was observed. Therefore, the optimal CF value was set at 1.6 ml min^–1^. The final conditions of the optimised AF4 separation method for TiO_2_ were fixed at: Ft, 5 min; CF_exp_, 0.6%; DF, 0.4 ml min^–^^1^; and CF, 1.6 ml min^–1^.

### Stability of the sample

**Figure 5.**
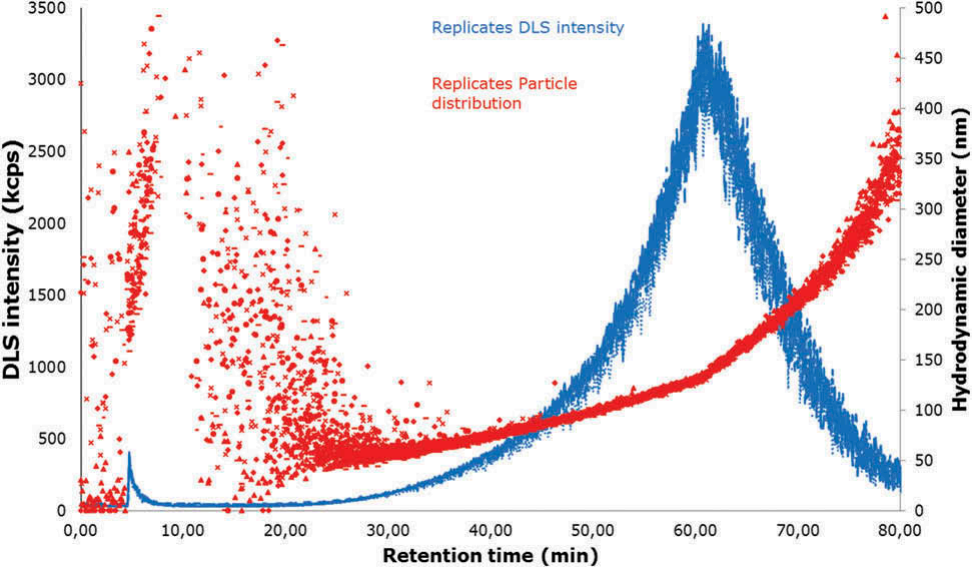
(colour online) Dynamic light scattering (DLS) intensity (blue) and particle size distribution (hydrodynamic diameter; red) plotted versus time obtained for 10 replicate measurements of JRCNM0-1004a by applying the optimised AF4 protocol and performed over 24 h. The different curves and distributions coincided very well, thereby demonstrating the very good repeatability of the measurement system and the stability of the sample over 24 h.

Once the best working conditions of the AF4 system were acquired, the stability of the sample was investigated. When applying this technique in a reproducible way, the particle dispersion has to be stable during the time of the analysis. The stability of the sample was studied as performance characteristics of the selected dispersion protocol by performing 10 replicate separations of the same sample covering a time span of 24 h. The results of analysis are shown in Figure 5, where the DLS response is plotted for all 10 measurements, and no appreciable decrease in intensity could be observed, indicating that the sample, as well as the separation system, is stable during that time. Thus, the dispersion protocol is fit for purpose.

### Sample size distribution of JRCNM0-1004a

**Figure 6.**
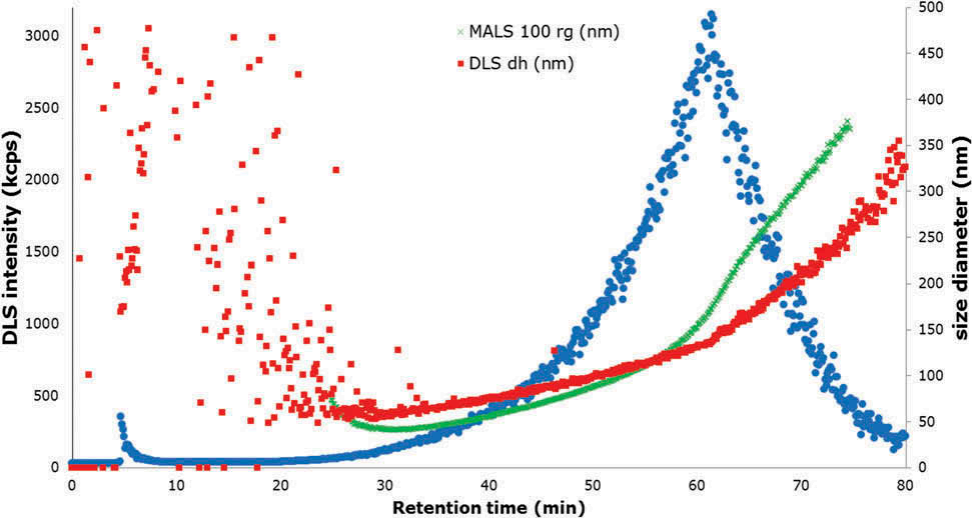
(colour online) Fractogram obtained from the AF4-MALS-DLS, DLS signal (blue) showing the elution profile of the JRCNM0-1004a; *d*_h_, hydration diameter obtained by DLS (red squares); *r*_g_, radius of gyration obtained by MALS at an angle of 100° converted into diameter (green crosses).

When AF4 is coupled to a DLS and an MALS, a description of the particle shape can be obtained by calculating the *r*_g_/*r*_H_ (the ratio of the radius of gyration and radius of hydration) (Lohrke et al. 2008). In this case, the particle size distribution was measured using AF4 online with MALS and DLS. The particle size distribution range measured with both instruments was in the linear range of the fractogram, as shown in Figure 6, which means the particles are eluting according to their size in a linear way.

The DLS detector provides the hydrodynamic diameter of the particles; and MALS gives the radius of gyration. The particle size distribution was estimated from minutes 26 to 65. As can be observed in Figure 6, before minute 26 there were still particles from the void peak eluting and it has to be also considered that after minute 65 the cross-flow was set to zero and the particles that were still in the system were released. The measured size distribution in particle diameter was from 50.5 ± 0.8 to 197.9 ± 3.2 nm. The values obtained with the DLS agree with statements in Rasmussen et al. (2014).

With the MALS detector, the integrated area for the estimation of the particle size distribution was between minute 30 – because the linear increasing range starts at that moment – and minute 65, as shown in Figure 6. The corresponding radius of gyration was 14.7 ± 0.4–157.0 ± 1.5 nm, calculated by the algorithm Random Coil, which assumes arbitrary particle sizes. AF2000 Control software allows the user to choose among different models (Zimm, Berry, Debye, Hollow Sphere, Random Coil, Relative) in order to calculate the radii of the particles. In this case, where titanium is polydisperse, it was observed that Random Coil was the algorithm that fits better with all the different angles of the MALS, thus giving robustness to the method (von der Kammer et al. 2005).

The results from both detectors were also utilised to estimate the shape of the particles present in the sample. This was possible since according to Lohrke et al. (2008) different shapes of particles show a different ratio (*r*_g_/*r*_H_) of the particle size obtained with both detectors. For instance, spherical particles have an *r*_g_/*r*_H_ between 0.775 and 1, while rods tend to have a ratio > 2 (Lohrke et al. 2008). In the analysed polydisperse material, two different groups of particles could be identified since for up to about 50 min the slopes of linear curves from both detectors coincided, whereas above this retention time the slope of the MALS curve was steeper compared with the DLS curve. This led to different ratios below and above 50 min, indicating that below this limit most of the particles seemed to be spherical (*r*_g_/*r*_H_ ~ 0.7), whereas after this limit mainly later random shaped particles were eluting (*r*_g_/*r*_H_ ~ 1.5).

The recovery of the optimised method was calculated by injecting 20 µl of the sample with the cross-flow set to zero and calculating the area under the curve of the UV-Vis (Geiss et al. 2013; Zhou & Guo 2015). Next, another 20 µl of the same sample were injected under the optimised conditions, in which the sum of the area of the void peak and the main peak (together with the corresponding release peak, which elutes after minute 65 when the CF is stopped) were estimated in the UV-Vis and the recovery was calculated by dividing the areas of interest:

(1) 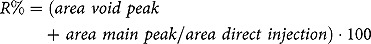

Von der Kammer et al. (2011) recommend that the quality of the separation is evaluated by sample recovery and that ideally it should be calculated for more than one detector. For the studied material, the recovery was 73 ± 3% (standard error of the mean, *N* = 5) for the DLS and 62 ± 8% for the UV-Vis. The complexity of a polydisperse material means it is considered an acceptable recovery for the developed method. As mentioned above, a blank run had to be done after each experiment to avoid carryover effects; this affects directly the estimation of the recovery. Moreover, it is known that the use of salt can help keep the particles disaggregated (Brar & Verna 2011; Mudalige et al. 2015), though it could also push them to the membrane; thus, a loss of the sample could be expected.

## Conclusions

This study developed an analytical method for the characterisation of coated polydisperse TiO_2_ nanoparticles. The method comprised two steps: sample preparation with focused ultrasound and AF4 coupled to DAD, DLS and MALS detectors. Both steps required the optimisation of the experimental conditions, which was pursued in this study using experimental designs. The outcome of the study demonstrated that this approach allowed the identification of optimal experimental conditions, while the required number of experiments was minimised. Finally, the optimised method protocol was applied to replicate analyses of polydisperse TiO_2_, showing acceptable values for repeatability (< 3%) and recovery (> 60%).

The same approach employed in this study was successfully applied to the characterisation of several non-coated and commercially available polydisperse TiO_2_ materials, some authorised and used as feed additives in the European market. These results, together with the assessment of a number-based size distribution of these materials which is being carried out at the moment, will be presented in a second study.
